# Multi- and Transgenerational Effects of Developmental Exposure to Environmental Levels of PFAS and PFAS Mixture in Zebrafish (*Danio rerio*)

**DOI:** 10.3390/toxics10060334

**Published:** 2022-06-18

**Authors:** Alex Haimbaugh, Chia-Chen Wu, Camille Akemann, Danielle N. Meyer, Mackenzie Connell, Mohammad Abdi, Aicha Khalaf, Destiny Johnson, Tracie R. Baker

**Affiliations:** 1 Department of Pharmacology, Wayne State University, Detroit, MI 48202, USA; alexhaim@wayne.edu (A.H.); chiachenwu@ufl.edu (C.-C.W.); gi2263@wayne.edu (C.A.); danielle.meyer@ufl.edu (D.N.M.); 2 Institute of Environmental Health Sciences, Wayne State University, Detroit, MI 48202, USA; gg8277@wayne.edu (M.C.); mohammed.abdi@wayne.edu (M.A.); aichakhalaf@wayne.edu (A.K.); destinyjohnson@wayne.edu (D.J.); 3 Department of Environmental and Global Health, University of Florida, Gainesville, FL 32610, USA

**Keywords:** PFAS, PFAS mixtures, epigenetics, zebrafish, transgenerational

## Abstract

Per- and polyfluoroalkyl substances (PFASs) are ubiquitous in the environment and are tied to myriad health effects. Despite the phasing out of the manufacturing of two types of PFASs (perfluorosulfonic acid (PFOS) and perfluorooctanoic acid (PFOA)), chemical composition renders them effectively indestructible by ambient environmental processes, where they thus remain in water. Exposure via water can affect both human and aquatic wildlife. PFASs easily cross the placenta, exposing the fetus at critical windows of development. Little is known about the effects of low-level exposure during this period; even less is known about the potential for multi- and transgenerational effects. We examined the effects of ultra-low, very low, and low-level PFAS exposure (7, 70, and 700 ng/L PFOA; 24, 240, 2400 ng/L PFOS; and stepwise mixtures) from 0–5 days post-fertilization (dpf) on larval zebrafish (*Danio rerio*) mortality, morphology, behavior and gene expression and fecundity in adult F0 and F1 fish. As expected, environmentally relevant PFAS levels did not affect survival. Morphological abnormalities were not observed until the F1 and F2 generations. Behavior was affected differentially by each chemical and generation. Gene expression was increasingly perturbed in each generation but consistently showed lipid pathway disruption across all generations. Dysregulation of behavior and gene expression is heritable, even in larvae with no direct or indirect exposure. This is the first report of the transgenerational effects of PFOA, PFOS, and their mixture in terms of zebrafish behavior and untargeted gene expression.

## 1. Introduction

Per- and polyfluoroalkyl substances (PFASs) are a class of chemicals constituted by a polar head group attached to a chain of C-F bonds. The unique chemistry of these compounds renders them effectively indestructible and, thus, a prime candidate for high-heat industrial processes and long-lasting consumer goods such as non-stick cookware and waterproofed outerwear. The utility of PFASs is offset by their bioaccumulation and toxic health effects. PFAS are detected virtually everywhere—in diverse wildlife, multiple environmental matrices, and in >99% of the general public [1,2,3]. Drinking water is a significant source of exposure in humans [4], and drinking water treatment plants are not designed to remove these contaminants from source water. Likewise, wastewater treatment plants do not intentionally filter out PFASs. PFASs are commonly found to be in the parts per trillion (ppt; ng/L) range in both untreated and treated drinking water [5] and wastewater [6]. The widespread low-level exposure warrants investigation into the health effects on wildlife and humans.

PFASs readily cross the placental barrier, potentially exposing a fetus during sensitive time periods during development [7]. Chemical assault during critical windows in development can have effects later in life; this thinking stems from the developmental origins of health and disease (DOHaD) hypothesis [8]. DOHaD posits that the timing of the exposure is crucial in determining the result. Placental transfer of PFASs necessitates the study of early-life exposure and the heritable effects of exposure. As most people have small amounts of many types of PFASs in their bodies, it is of great general interest to study the effects of low-level exposure, including to mixtures, on developing organisms.

The two most common PFASs carried by the general population are perfluorooctanoic acid (PFOA) and perfluorooctane sulfonic acid (PFOS), and they are usually detected at higher levels than other types of PFASs. PFOS is present at approximately three times the levels of PFOA in humans [9]. Choosing a relevant exposure concentration is of importance when planning translational experiments to realistically inform public health. The general population carries serum PFAS levels in the μg/L range (>999 ng/L), with an average of 1.42 μg/L PFOA and 4.25 μg/L PFOS [9]. The Environmental Protection Agency (EPA) health advisory limit for drinking water of PFOA, PFOS, or their combined concentration in mixture is 70 ppt, or 70 ng/L. Much early work characterizing PFAS toxicity used, understandably, high dose experiments to define outcomes, such as the concentration at which 50% of exposed organisms die (LC_50_). We now know that PFAS levels, while ubiquitous in all environmental compartments, are typically at ng/L or μg/L levels in water. A study of treated water from 25 drinking water treatment plants across the United States found a median concentration of 19.5 ng/L for 17 PFASs combined, with a maximum sum of 1.1 μg/L (1100 ng/L) [5]. Our group has previously shown that mean concentrations of PFOA and PFOS in a waterway that provides drinking water in a major metropolitan area in Michigan were 2.2 ng/L and 2.9 ng/L, respectively [10]. In order to advance public health knowledge of exposures at both environmentally relevant levels and levels encompassing the EPA health advisory, we chose exposure concentrations for PFOA of 7, 70, and 700 ng/L and for PFOS at approximately 3× higher concentrations of 24, 240, and 2.4 μg/L (2400 ng/L), a ratio similar to reported human levels. The mixture concentrations contain half of each exposure level per chemical (e.g., the ultra-low mixture concentration contains 3.5 ng/L PFOA and 12 ng/L PFOS). Throughout this report, we will refer to these nominal concentrations of 7 ng/L PFOA exposure and 24 ng/L PFOS exposure as the “ultra-low” exposure level, the 70 ng/L PFOA and 240 ng/LPFOS exposure as “very low”, and the 700 ng/L PFOA and 2.4 μg/L PFOS exposure as “low”. Exposures in other studies within the ng/L range are referred to as “low”, μg/L range as “moderate”, mg/L range as “high”, and g/L range as “very high”.

As individual PFASs are seldom discovered in the environment or treated drinking water alone, it is critical to study mixtures at environmental levels. PFAS mixtures are increasingly studied, but their effects are still unclear and often unpredictable, especially at different concentrations. Ding et al. [11] characterized the 1:1 mixture of PFOA and PFOS at high concentrations to be synergistic towards early-life lethality in zebrafish, while increasing the PFOA:PFOS ratio resulted in antagonism, then additivity. In another study, individual PFASs alone significantly changed swim behavior in exposed fish at moderate levels, but a mixture of nine PFAS had no effect at environmental levels [12]. We sought to address this gap by characterizing a low-level mixture of the two most commonly detected PFAS.

There is emerging evidence that PFAS exposure confers heritable effects on later generations via epigenetic mechanisms [13] rather than direct genotoxicity. Epigenetic modifications to DNA or chromatin serve as a “biological memory” of environmental history that modulate gene regulatory networks in current and future generations [14]. Toxicoepigenetic initialization in the directly exposed organism can be perpetuated across multiple generations. When an individual is directly exposed, the exposure indirectly affects germ cells residing in the individual. “Multigenerational” (F1) effects are seen in the generation following the directly exposed (F0) generation. Even if the exposure ceases, indirect germ cell exposure has occurred and can present phenotypically in this next generation’s life. Effects are considered “transgenerational” when observed in the subsequent (F2) generation, which has never been directly or indirectly exposed. Zebrafish exposures with explicit epigenetic outcomes through multiple generations have not been conducted for PFAS. However, in the F0 generation, Bouwmeester et al. [15] found that moderate-range PFOA exposure increased methylation associated with *vtg1*, a gene involved in fertility. Limited epigenetic studies have been done in rodents. Tian et al. (2019) found that non-specific methylation therapy administered with PFOS to F0 females resulted in better birth outcomes in F1 pups than F0 PFOS exposure without methylation therapy [16]. The potential heritability of PFAS exposure effects is pertinent, as measures taken now to prevent or reduce exposure could magnify public health benefits to the next generation(s) at scale. The results of the current study suggest that epigenetic mechanisms mediate each generation’s response to exposure in terms of behavior and gene expression.

The zebrafish is an ideal model system for conducting early-life research on water-borne contaminants over multiple lifetimes. Zebrafish have been a useful, popular model in developmental toxicology due to their easy visibility, high *n*-values, quick generation time, and high homology with the human genome [17]. Additionally, the EPA plans to eliminate funding for mammalian vertebrate research completely by 2035 [18], positioning the zebrafish as a pertinent alternative model organism. From the outset, zebrafish eggs have a transparent chorion through which development can be observed from the single-cell stage to free-swimming larvae at five days post-fertilization (5 dpf). Zebrafish are prolific breeders, producing > 300 eggs per week, and are sexually mature in ~3 months [19], meaning transgenerational effects can be observed in about one year. They have been utilized as an ideal transgenerational model due to all of the above-mentioned benefits and the external fertilization of eggs, which reduces the number of generations compared to mammalian models [20].

This study aims to advance understanding of the short- and long-term health effects of developmental exposure to environmentally relevant levels of PFASs using the zebrafish model organism. After exposing embryonic zebrafish to environmental levels of two prevalent PFASs, PFOS and PFOA, and a mixture of the two chemicals (referred to throughout simply as “mixture”) for the first 5 days of life, we found that swimming behavior and gene expression at 5 days post-fertilization (dpf) was affected by at least one concentration of all chemicals in all three generations (F0–F2). Pathway analysis of gene expression revealed upregulated pathways of immunotoxicity, movement disorders, and endocrine disruption. Adult fecundity (eggs produced per female) was statistically increased in the PFOA-exposed F0 generation and decreased in the F1 generation. Morphological abnormalities at 5 dpf were not observed until the F1 and F2 generations. As expected at these low doses, survival was uniformly unaffected by exposure.

It is the authors’ aim that these results inform decision-making regarding safe contaminant limits in drinking water and in aquatic habitats. The federal health advisory limit set by the EPA for PFOS, PFOA, and their mixture is currently 70 ng/L [21], while some states legislate much lower levels. This study provides the first report of multigenerational effects of PFOA exposure on behavior and of mixture exposure on behavior and gene expression, supporting findings in other PFOS studies showing these endpoints are affected multigenerationally. Further, we show novel transgenerational effects on behavior and gene expression following low-level exposure to any PFAS during early life. Future efforts should include complex mixtures, and PFAS replacements, including “short-chain” alternatives, will be critical to study as well.

## 2. Materials and Methods

### 2.1. Animal Husbandry of Adult Fish

Adult AB strain zebrafish were maintained on a 14:10 h light:dark cycle, as previously described [22], on a recirculating system of RO water buffered to a neutral pH with Instant Ocean© salts (Spectrum Brands, Blacksburg, VA, USA) at 27–30 °C. Ammonia and nitrite levels remained at 0 ppm. Fish were fed twice daily (Aquatox Fish Diet, Zeigler Bros Inc., Gardners, PA, USA) and supplemented with brine shrimp (Artemia International, Fairview, TX, USA). All zebrafish use protocols were approved by the Institutional Animal Care and Use Committee at Wayne State University, according to the National Institutes Health Guide to the Care and Use of Laboratory Animals (Protocol 16-03-054; approved 4 August 2016).

### 2.2. PFAS Exposures

#### 2.2.1. Spawning Procedure 

To obtain F0 embryos, adult stock zebrafish were spawned in a 2:1 female:male ratio (at least 4 trios per concentration) (Figure S1) in the environmental conditions described above. Sexes were separated overnight by a plastic divider in a spawning tank and were allowed to spawn at 08:00 the next morning. Spawning tanks contained a slotted insert through which eggs fell to the bottom, away from the adults. Embryos were harvested after 2 h of spawning activity. 

#### 2.2.2. Egg Cleaning

Eggs were incubated at 27 °C in 58 ppm bleach for 10 min, rinsed with RO water, and then placed back in their normal environment of a weak salt solution (600 mg/L salt in RO water) containing Instant Ocean© salts (Spectrum Brands, Blacksburg, VA, USA).

#### 2.2.3. Exposure Protocol 

Perfluorooctanoic acid (PFOA) (CAS# 335-67-1, Sigma, St. Louis, MO, USA, 95% purity) and perfluorooctane sulfonic acid (PFOS) (CAS# 1763-23-1, Sigma, 99.4% purity) were used for stock solutions. From these stock solutions, serial dilutions in RO water buffered with Instant Ocean© salts were carried out each day of the exposure to reach the nominal concentrations of 7, 70, and 700 ng/L for PFOA; 24, 240, and 2400 ng/L PFOS; and a mixture with half of the individual concentrations and 1:1 volume ratios (e.g., the ultra-low mixture concentration would contain 3.5 ng/L PFOA and 12 ng/L PFOS). The control was exposed to RO water buffered with Instant Ocean salts; 30 embryos (≤4 hpf) were placed into a well of a 6-well Falcon plate with 8.5 mL of their respective chemical concentration or buffered water (controls). Solutions were replenished daily with approximately 90% fresh solution. Larvae were maintained in an incubator at 27 °C. On day 5, all larvae were rinsed three times in buffered water solution to end the exposure before proceeding with further assays.

### 2.3. Survival and Abnormality Screening

Survival was recorded on day 5 post-fertilization. Embryos or larvae were considered dead if the heart was stopped. On day 5, all hatched survivors were screened via light microscope for cardiac edema, yolk sac edema, presence of swim bladder, and bent spine. Student’s *t*-test was used to determine the statistical significance of each concentration compared to control in terms of the percent total abnormalities. Assays were repeated a minimum of 5 times, with at least 150 larvae per concentration (Table S1). Each repetition was performed on a different day with different larvae.

### 2.4. Behavioral Analysis

The behavioral assay measuring swim distance in light and dark cycles was performed and analyzed as previously reported [20]. Briefly, healthy (no morphological abnormalities) 5 dpf larvae from control and exposed groups were acclimated to a well plate for ≥1 h, then loaded into a DanioVision Chamber (Noldus Information Technology, Wageningen, The Netherlands), which alternated four light and dark cycles for three min each following a chamber acclimation period. Raw data were exported to Noldus EthoVisionXT14, and average distance moved (cm) was analyzed using ANOVA and Tukey’s HSD tests in custom R scripts (File S1). The assay was replicated at least three times for each chemical or mixture, with at least 68 fish per concentration in each replicate (Table S1). Each repetition was performed on a different day with different larvae. Larvae were euthanized after the behavioral assay and not used for any further endpoints.

### 2.5. RNA-Seq and Pathway Analysis

At 5 dpf, five larvae were euthanized and pooled to create one sample, and at least 3 samples per concentration were analyzed for gene expression (Table S1). Each repetition was performed on a different day with a different cohort of larvae. Larvae were pooled to represent the ratio of healthy:abnormal larvae observed during the morphological abnormality assay. For example, if 20% of all low-level PFOA larvae presented abnormalities, 1 of each 5 pooled larvae would present an abnormality, while the other 4 were healthy. Larvae, once euthanized in 16.7 mg/mL tricaine methanesulfonate, were placed in RNALater. This was drained according to the manufacturer’s instructions (i.e., between 1–7 days later) and then stored at −80 °C. Storage of larvae, RNA isolation, cDNA library preparation, sequencing, differential expression analysis, and pathway analysis were performed as previously reported [22]. Briefly, RNA isolation was performed with the Qiagen RNeasy Lipid Mini Kit (Qiagen, Hilden, Germany). cDNA libraries were prepared using the Quantseq™ 3′ mRNA-seq kit (Lexogen, Vienna, Austria). RNA and cDNA concentrations were measured with a Qubit™ 2.0 fluorometer (Invitrogen, Carlsbad, CA, USA), and cDNA quality was also assessed with an Agilent TapeStation 2200 (Agilent Technologies, Santa Clara, CA, USA). F0 samples were sequenced on Illumina^®^ MiSeq™ (Illumina, CA, USA) and F1–F2 were sequenced on Illumina^®^ HiSeq 2500™ (Illumina, CA, USA) using the Lexogen Bluebee^®^ Genomics Platform (Bluebee, Rijswijk, The Netherlands). F0 reads were aligned to *Danio rerio* genome Build GRCz10, and F1–F2 reads were aligned to *Danio rerio* genome Build GRCz11; differential expression analysis was determined via DESeq2. Differentially expressed genes (DEGs) with log2 fold changes ≥0.75 or ≤−0.75, *p*-values <0.01, and ≥50 analysis-ready molecules were analyzed with Ingenuity Pathway Analysis (IPA^®^) software (Qiagen Bioinformatics, Redwood City, CA, USA).

### 2.6. Fecundity Assay

At sexual maturity and dimorphism (4–6 weeks), fish were spawned in a 1:1 male:female ratio in order to attribute the number of eggs produced to each individual female (Figure S1). Fish were not spawned more than once per week. Four randomly-chosen pairs per concentration and control were used per experiment (16 total spawning tanks) (Figure S1, Table S1). After two acclimation sessions of spawning, experiments were replicated a minimum of three times, and a minimum of 6 clutches per concentration were analyzed. Males and females were separated overnight by a plastic divider. At 08:00, dividers were removed and spawning allowed for 2 h. Then, each clutch was cleaned (as described in Section 2.2.2), and eggs were imaged for later quantification. Student’s two-tailed *t*-test was used to determine the average number of eggs per female for each concentration and chemical.

### 2.7. Sex Ratio

At maturity, fish were visually assessed for female or male secondary sex characteristics. Chi-squared tests were used to determine the statistical significance of any concentration compared to control (Table S1). In F0 fish, dissection was performed for validation. Fish were euthanized in 1.67 mg/mL tricaine methanesulfonate (Syndel, Ferndale, WA, USA) for 10 min.

## 3. Results

### 3.1. F0 Generation

Table 1 shows significant endpoints in all chemicals and concentrations.

#### 3.1.1. F0 Survival and Abnormalities

No statistically significant larval abnormalities or mortality were observed in any concentration of any chemical or mixture. Ultra-low PFOS exposure approached significance with a slightly higher rate of abnormalities (*p* = 6.3 × 10^−2^) (Table S1).

#### 3.1.2. F0 Behavior

##### PFOA 

**Figure 1:**
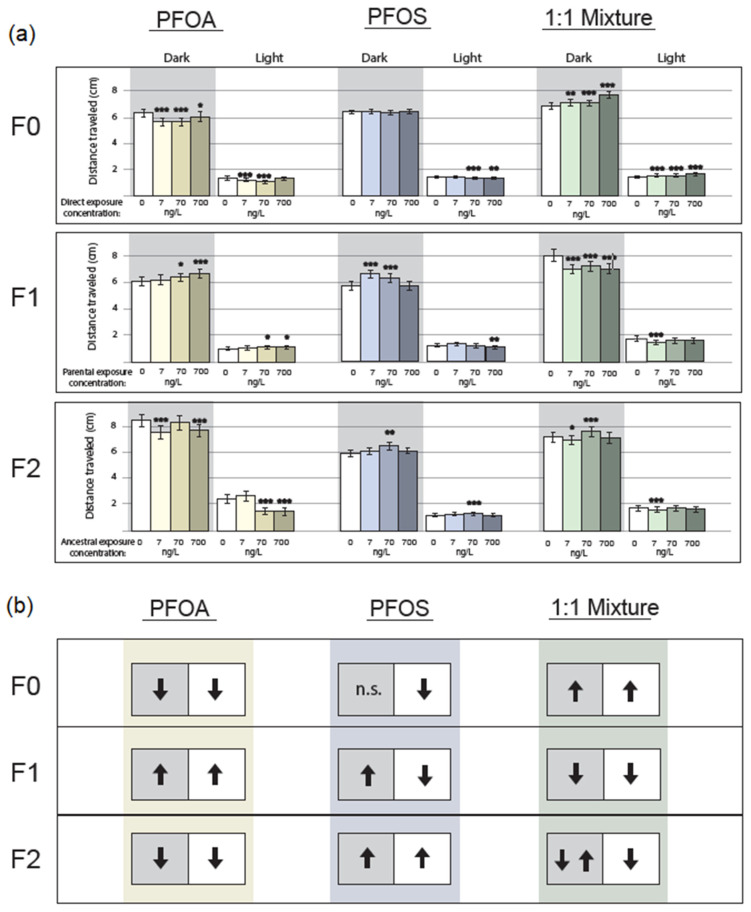
Locomotion following PFAS exposure in dark and light. Yellow: PFOA. Blue: PFOS. Green: mixture. (**a**) Top panel: F0 generation. Middle panel: F1 generation. Lower panel: F2 generation. * *p* < 0.05, ** *p* < 0.01, *** *p* < 0.001; ANOVA with Tukey pairwise test. 0: no exposure. UL: ultra-low exposure. VL: very low exposure. L: low exposure. (**b**) Simplified representation of significant behavioral direction. Upwards arrow: hyperactivity. Downwards arrow: hypoactivity. Two arrows: discordance between one or more concentrations on hyper- vs. hypoactivity. n.s.: not significant.

**Figure 2:**
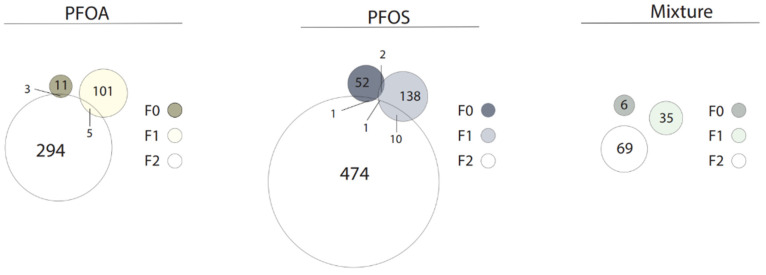
Number of DEGs in each generation for each chemical.

Direct PFOA exposure significantly decreased larval swimming distance in both dark and light cycles at every concentration, with the exception of the low concentration in the light (Figure 1 and Figure 2) (*p* < 1 × 10^−8^; *p* <1 × 10^−8^; *p =* 2.1 × 10^−2^; ultra-low, very low, low exposure in the dark, respectively) (*p =* 3.4 × 10^−4^; *p* < 1 × 10^−8^; ultra-low and very low exposure in the light, respectively).

##### PFOS

Direct PFOS exposure had no effect on larvae from any concentration in the dark. In the light, very low and low exposure groups were significantly hypoactive (Figure 1 and Figure 2) (*p =* 4 × 10^−7^, 1.2 × 10^−3^, respectively). 

##### Mixture

Direct exposure to the mixture of PFOA and PFOS resulted in increased swimming distance in larval zebrafish (*Danio rerio*), regardless of light/dark setting (Figure 1 and Figure 2) (dark: *p =* 1.6 × 10^−3^; *p =* 8.1 × 10^−4^; *p* < 1 × 10^−8^; ultra-low, very low, low exposure, respectively) (light: *p =* 6.7 × 10^−4^; *p =* 6.4 × 10^−5^; *p* < 1 × 10^−8^; ultra-low, very low, low exposure, respectively). 

#### 3.1.3. F0 Transcriptomics 

**Figure 3:**
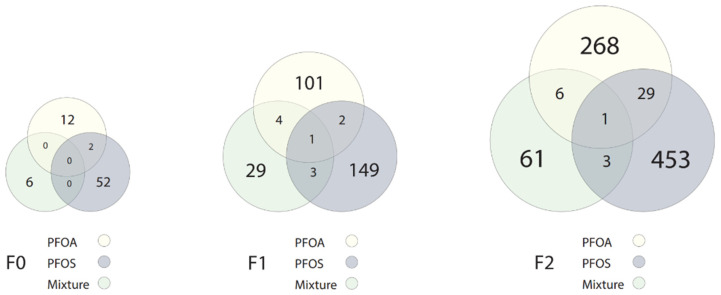
Number of DEGs from each chemical in every generation. Size of the circle indicates the proportion of genes expressed; color of the circle indicates the chemical.

The full lists of DEGs for all chemicals and concentrations in the F0 generation can be found in Table S3; the top five up- and downregulated DEGs are shown in Table 2. Venn diagrams illustrating the overlap of generation-specific DEGs (all concentrations combined) are in Figure 2. Venn diagrams illustrating the overlap of F0 DEGs for each chemical (all concentrations combined) are in Figure 3. DEGs are considered significant at *p* < 0.01 and log2FC of ≥0.75 or ≤−0.75. Pathway analysis could not be performed due to an insufficient number of DEGs.

##### PFOA 

Exposure to the ultra-low level of PFOA had no effect on differential gene expression. At very low exposure, only *rpe65a* was significantly changed (LFC −0.89, *p =* 9.7 × 10^−10^). Basic cellular functions were impacted by low exposure. Of the 14 genes that were differentially expressed (DEGs) (11 up, 3 down), *tmem14c* was the most upregulated (LFC 0.96) and *atp6v0e1* the most downregulated (LFC −0.85).

##### PFOS 

Exposure to the ultra-low level of PFOS significantly increased the expression of the insulin receptor substrate *irs2a* (LFC 0.77). Of 54 DEGs, the inflammatory response gene *irg1l* was the most upregulated following very low exposure (LFC 1.72); the most downregulated was the kinesin *kif3c* (LFC −1.24). Low exposure to PFOS did not elicit gene expression changes.

##### Mixture

Exposure to ultra-low levels of PFAS mixture significantly downregulated six genes. The most downregulated was the isomerase *fkbp9* (LFC −0.92). Very low exposure had no effect on gene expression. Low exposure induced the upregulation of two genes, the amino acid transporter *slc6a19a.1* (LFC 0.82) and the nucleoside biosynthesis gene *entpd8* (LFC 0.78).

#### 3.1.4. F0 Fecundity

##### PFOA 

Early-life PFOA exposure did not significantly affect adult female egg production at any concentration (Figure S2). Full fecundity data are shown in Table S2.

##### PFOS

Fecundity trended downwards with increasing concentrations of PFOS but did not reach statistical significance (low exposure: *p =* 7 × 10^−2^) (Figure S2). Full fecundity data are shown in Table S2.

##### Mixture

Early-life mixture exposure did not significantly affect fecundity at any concentration (Figure S2). Full fecundity data are shown in Table S2.

#### 3.1.5. F0 Adult Body Weight/Length

Adult body weight or length was unaffected by any concentration of any chemical significantly. Females exposed to PFOA at very low and low levels trended towards being significantly heavier (*p =* 5.1 × 10^−2^, 6.5 × 10^−2^, respectively); 15–17 fish were evaluated per concentration, with one replicate per exposure group.

#### 3.1.6. F0 Sex Ratio

No chemical or concentration affected the sex ratio in the F0. Low mixture exposure approached a significant decrease in the male:female ratio (0.73, *p* = 5.3 × 10^−2^, *n* = 22–23). The control male:female ratio for PFOA was 1.11 (*n* = 19–25), for PFOS was 2.09 (*n* = 29–34), and for the mixture was 2.67; 19–34 fish were evaluated per concentration, with one replicate per exposure group.

### 3.2. F1 Generation

Table 1 shows significant endpoints for all chemicals and concentrations.

#### 3.2.1. F1 Abnormalities and Survival

No statistically significant abnormalities or mortality were observed in any concentration of PFOS or the mixture. At low PFOA exposure, a significant decrease was observed in abnormalities (*p* = 2.9 × 10^−2^) (Table S1), and at ultra-low exposure, a significant increase was observed for survival (*p* = 3.6 × 10^−4^).

#### 3.2.2. F1 Behavior

##### PFOA 

Parental PFOA exposure was associated with increased swimming activity in both light and dark at the very low and low concentrations (Figure 1 and Figure 2) (dark: *p =* 1.3 × 10^−2^; *p* < 1 × 10^−8^; 70 and 700 ng/L, respectively) (light: *p =* 4 × 10^−2^, 1.3 × 10^−2^, respectively). 

##### PFOS

Parental PFOS exposure was associated with increased larval activity in the dark at the very low and ultra-low concentrations (*p* < 1 × 10^−8^ for both), yet activity decreased in the light at the low concentration (*p =* 5.9 × 10^−3^) (Figure 1 and Figure 2). 

##### Mixture

Parental exposure to the PFAS mixture strongly decreased swimming behavior in the dark at all concentrations (*p* < 1 × 10^−8^ for all) and in the light as well only at the ultra-low level (*p =* 3.4 × 10^−6^); *n* = 72 per concentration (Figure 1 and Figure 2).

#### 3.2.3. F1 Transcriptomics and Pathway Analysis

The full lists of DEGs for all chemicals and concentrations in the F1 generation can be found in Table S4; the top five up- and downregulated DEGs and affected pathways are shown in Table 2. The full lists of pathways (where applicable) for all chemicals, concentrations, and generations can be found in Table S5. Venn diagrams illustrating the overlap of generation-specific DEGs (all concentrations combined) are in Figure 2. Venn diagrams illustrating the overlap of F1 DEGs for each chemical (all concentrations combined) are in Figure 3. DEGs are considered significant at *p* < 0.01 and log2FC of ≥0.75 or ≤−0.75.

##### PFOA 

Parental PFOA exposure at the ultra-low level caused the significant upregulation of 12 genes and the downregulation of 5 genes (log2FC < −0.75). The most upregulated gene (log2FC = 1.03) was *dusp16* and the most downregulated *si:ch211-125e6.5* (log2FC: −0.84). At very low exposure, 106 genes were significantly differentially expressed (64 upregulated, 42 downregulated). The most upregulated gene was *dusp27* (log2FC: 1.24), and the most downregulated was the innate immunity-related *c3a.2* (log2FC: −1.44). The genes were involved in pathways of xenobiotic metabolism via the CAR pathway and estrogen receptor signaling. Other xenobiotic pathways involved were LXR, RXR, AhR, and FXR. The kinase *dusp27* was the most upregulated molecule. With low parental exposure, there were 49 DEGs, with the most highly upregulated gene of 37 genes being *tmigd1* (log2FC: 1.26) and the most downregulated *trmt1* (log2FC: −0.87), out of 12. 

##### PFOS

Parental PFOS exposure at the ultra-low level caused the significant upregulation of five genes. The most upregulated gene (log2FC = 0.96) was *satb1a*. Very low parental exposure resulted in only two significant DEGs: *npas4a* (log2FC: 0.75) and *slc43a2a* (log2FC: −0.94). Low parental exposure resulted in 149 significant DEGs. The most highly upregulated of 118 genes was *zmat5* (log2FC: 1.29); the most downregulated gene of 80 genes was *mfsd14ba* (log2FC: −1.14). Pathway analysis indicated increased lipid metabolism and decreased cell death pathways.

##### Mixture

Parental exposure to the ultra-low level of PFAS mixture induced 35 significant DEGs. Of the 21 upregulated genes, *zgc:92590* was the highest (log2FC: 1.35). Of the 13 downregulated genes, *smtnl* was the most downregulated (log2FC: −1.17). Very low parental exposure was associated with the upregulation of 11 genes, with *cela1.3* being the most upregulated (log2FC: 1.19), and only 1 downregulated gene (*panx1a*, log2FC: −0.85). At low parental exposure, five genes were upregulated, with *cpa4* the most upregulated (log2FC: 0.88), and two genes were downregulated: *rlbp1b* and *ms4a17a.8* (log2FC: −0.79, −0.89, respectively).

#### 3.2.4. F1 Fecundity

##### PFOA 

The F1 generation of very low-level PFOA exposure lineage produced significantly fewer eggs than controls (−28.2%, *p <* 0.01). Ultra-low and low concentrations were not affected (Figure S2). Full fecundity data are shown in Table S2.

##### PFOS

Parental PFOS exposure had no effect on F1 fecundity (Figure S2). Full fecundity data are shown in Table S2.

##### Mixture

Parental mixture exposure had no effect on F1 fecundity (Figure S2). Full fecundity data are shown in Table S2.

#### 3.2.5. F1 Sex Ratio

##### PFOA

At every concentration (ultra-low, very low, low), there was a significant increase in the male:female ratio of adult fish (*p =* 7.4 × 10^−4^, 2.6 × 10^−9^, 3.2 × 10^−9^, respectively). The authors note the abnormal lack of males in the control group, which may have led to a false-positive result (PFOA control male ratio: 0.17, PFOS control male ratio: 1.53, mixture control male ratio: 1.12). PFOA F1 contained significantly fewer males than PFOS and mixture F1 (*p* = 4.1 × 10^−4^, 4.24 × 10^−5^, respectively (chi-square test)). There was no difference in control PFOS and mixture male ratio (*p* = 0.59). Additionally, due to a lack of access to research animals during the SARS-CoV-2-related institutional shutdown, only one cohort of fish (*n* = 56–66) could be observed. 

##### PFOS

No sex ratio shift was observed (*n* = 36–86).

##### Mixture

At the very low exposure level, there was a significant increase in the male:female ratio (4.45 compared to 1.12 in controls, *p =* 1.8 × 10^−3^, *n* = 55–63). The authors note that due to a lack of access to research animals during the SARS-CoV-2-related institutional shutdown, only two cohorts of fish could be observed.

### 3.3. F2 Generation

Table 1 shows significant endpoints for all chemicals and concentrations.

#### 3.3.1. F2 Abnormalities and Survival

No statistically significant abnormalities or mortality were observed in any concentration of any chemical or mixture.

#### 3.3.2. F2 Behavior

##### PFOA 

Transgenerational behavioral effects of legacy PFOA exposure manifested as hypoactivity at each concentration (Figure 1 and Figure 2) (ultra-low: *p* < 1 × 10^−8^ (dark); very low: *p* < 1 × 10^−8^ (light); low: *p =* 1 × 10^−2^ (dark); *p* < 1 × 10^−8^ (light)). 

##### PFOS

Transgenerational behavioral effects of legacy PFOS exposure manifested at only the very low exposure concentration, where hyperactivity was observed (Figure 1 and Figure 2) (dark: *p =* 7.6 × 10^−3^; light: *p* < 1 × 10^−8^). 

##### Mixture 

Transgenerational behavioral effects of legacy exposure to the PFAS mixture presented as hypoactivity following ultra-low exposure (Figure 1 and Figure 2) (dark: *p =* 4.2 × 10^−2^; light *p =* 3.2 × 10^−4^) and hyperactivity only at the very low concentration and only in the dark (*p* < 1 × 10^−8^). 

#### 3.3.3. F2 Transcriptomics and Pathway Analysis

The full lists of DEGs for all chemicals and concentrations in the F2 generation can be found in Table S5; the top five up- and downregulated DEGs and affected pathways are shown in Table 2. The full lists of pathways (where applicable) for all chemicals, concentrations, and generations can be found in Table S6. Venn diagrams illustrating the overlap of generation-specific DEGs (all concentrations combined) are in Figure 2. Venn diagrams illustrating the overlap of F2 DEGs for each chemical (all concentrations combined) are in Figure 3. DEGs are considered significant at *p* < 0.01 and log2FC of ≥0.75 or ≤−0.75.

##### PFOA

Ultra-low-level ancestral exposure to PFOA resulted in 112 significant DEGs in the F2 generation (30 upregulated, 82 downregulated). The most upregulated gene was *amy2al2* (log2FC: 4.41) and the most downregulated was *ms4a17a.8* (log2FC: −2.21). Pathway analysis revealed dysregulation of mitochondrial membrane potential and increased organismal injury, including cancer. In total, 106 DEGs resulted from ancestral PFOA exposure at the very low level (38 upregulated, 68 downregulated). As with the ultra-low concentration, the most upregulated gene here was the carbohydrate-metabolism-related *amy2al2* (log2FC: 3.58); the most downregulated was the sodium channel gene *scn2b* (log2FC: −2.62). Pathway analysis implicated cholesterol synthesis via CYP51A1 and other canonical pathways of sterol synthesis. Ancestral low PFOA exposure resulted in 302 significant DEGs in the F2 generation (124 upregulated, 178 downregulated). The most upregulated gene was again *amy2al2* (log2FC: 4.22), and the most downregulated was *lhx2b* (log2FC: −2.10). Pathways of immune function were upregulated; the top five most upregulated pathways all regard the trafficking of various immune cell types; 9 of the top 20 most upregulated pathways also feature cellular movement, 7 of these in immune cells specifically. The 20 most downregulated pathways feature 9 cell-death-related functions and 5 involved in the dysregulation of glucose homeostasis. Comparisons with GSEA datasets concerning epigenetic and/or chromatin regulation returned multiple DEGs (Table 3).

##### PFOS

Ultra-low-level ancestral exposure to PFOS resulted in 484 significant DEGs in the F2 generation (209 upregulated, 275 downregulated). The most upregulated gene was *cela1.5* (log2FC: 2.07), and the most downregulated was *pgk1* (log2FC: −2.34). Pathway analysis shows increased lipid metabolism, with 8 of the top 20 most upregulated pathways having to do with the synthesis or metabolism of steroids and terpenoids. The most downregulated pathway was bone mineral density (bias-corrected z-score: −2.75); other connective tissue pathways were overrepresented in the 20 most downregulated pathways. In total, 23 DEGs resulted from ancestral PFOS exposure at the very low level (16 upregulated, 7 downregulated). As with the ultra-low concentration, the most upregulated gene here was *cela1.5* (log2FC: 1.52); however, the most downregulated was *b3gntl1* (log2FC: −0.87). Ancestral low PFOS exposure resulted in seven significant DEGs in the F2 generation (four upregulated, three downregulated). The most upregulated gene was an unnamed/unannotated gene on chromosome 3 (*ENSDARG00000115830*) (log2FC: 1.38), and the most downregulated was *cfp* (log2FC: −1.00). Comparisons with GSEA datasets concerning epigenetic and/or chromatin regulation returned multiple DEGs (Table 3).

##### Mixture

Ultra-low-level ancestral mixture exposure was associated with 69 significant DEGs in the F2 generation. Of the 27 upregulated genes, *fzd6* had the highest log2FC (1.31). Of the 42 downregulated genes, *tcap* had the lowest log2FC (−1.19).

At the very low concentration, F2 larvae exhibited only one significantly upregulated DEG (*purab*, log2FC: 0.76) and four downregulated. The most downregulated gene was *hbae5* (log2FC: −1.36).

Similarly, the low level exposure had few DEGs. Two were upregulated (*fgfbp2b* and *si:dkey-102c8.3*, log2FC: 0.77, 0.76, respectively), and seven were downregulated (most downregulated: *si:ch211-281l24.3*, log2FC: −0.93). Comparisons with GSEA datasets concerning epigenetic and/or chromatin regulation returned one DEG, *h2bc1* (Table 3). No generation alone produced a sufficient number DEGs for pathway analysis; however, when all generations and concentrations were collated, pathways of cell death and immune dysfunction emerged (Table 4).

## 4. Discussion

In this study, numerous endpoints were examined across three generations of zebrafish exposed to environmentally relevant PFAS concentrations. Locomotion, gene expression, and fecundity were significantly altered across all generations by at least one concentration of PFOA, PFOS, and/or their mixture.

Environmental levels of PFAS exposure, as expected, did not cause significant mortality in any generation. Mortality with PFAS exposure is typically not observed in zebrafish under 10 mg/L (10^7^ ng/L) [23,24]. Jantzen et al. [25] also found no significant death or abnormalities using similar exposure methods to PFOS and PFOA. Gross morphological abnormalities were not increased by exposure, agreeing with literature noting abnormalities following ≥1 mg/L (10^6^ ng/L) PFAS exposure [26]. In the F1 generation of PFOA larvae, decreased abnormalities and increased survival were observed in the low and ultra-low groups, respectively. It is possible these unexpected outcomes may be due to exposure solutions, which were carefully derived from commercially available certified stock solutions but were not analytically verified, which may lead to variability in dosing; additionally, neither stock solution was available at 100% purity, ranging from 95% (PFOA) to 99.4% (PFOS) purity. Impurities of unknown origin, constituting up to 5% of PFOA exposure (0.35, 3.5, and 35 ng/L of the ultra-low, very low, and low concentrations, respectively) and 0.6% of PFOS exposure (0.14, 1.4, and 14 ng/L), could potentially have influenced the results. Overall, these results do not point to a severe risk of bodily harm from environmental-level exposure, though analytical verification of our exposure doses would support a higher-confidence assessment.

A persistent endpoint across all chemicals and generations was alterations in the behavioral response to light and dark stimuli. Larval swimming behavior is used as an indicator of neurotoxicity [27]. By 5 dpf, all major organ systems, including the brain, are functional [27]. Larvae are naturally more inclined to reserve swim bouts for dark periods, where they are less susceptible to predators than in the light [28]. Exposure-induced excitability or lethargy may be modulated by CNS function, which could translate to negative health implications in humans, and erratic behavior could have ecological consequences in aquatic wildlife consistently exposed to PFASs. We report multigenerational behavioral effects in the F1 generation and report for the first time transgenerational PFAS-associated behavioral changes in the completely unexposed F2 generation. The presence of a behavioral phenotype in the F2 generation suggests epigenetic changes induced by F0 exposure. More research is needed to plot the mechanism of this phenotypic inheritance, as well as how animal and human health and ecology are affected by continued PFAS exposure over multiple generations.

PFOA-exposed F0 larvae were hypoactive in both light and dark; this pattern was reversed in the indirectly exposed F1 generation, then returned to the F0 pattern in the unexposed F2 generation. Hyperactivity is typically seen in moderately to highly exposed F0 larvae [12,29,30]; however, exposure sometimes has no behavioral effect [31,32]. It is possible that PFOA exerts a non-monotonic response, wherein ng/L concentrations produce the observed hypoactivity, while higher doses produce hyperactivity. More research at low doses in the F0 generation will be required to draw conclusions. To our knowledge, this is the first study to examine behavior in the F1 and F2 generations of PFOA-exposed F0. The reversal in each generation of the direction of behavior (hypoactivity in F0; hyperactivity in F1; hypoactivity in F2) also suggests the possibility of a neuromodulatory compensation mechanism overcorrecting for the previous generation’s propensity for erratic behavior. 

PFOS-exposed F0 larvae were hypoactive in the light only; this persisted in the F1 generation. Additionally, F1 larvae were hyperactive in the dark, and F2 larvae were hyperactive under both conditions. In F0 larvae, hyperactivity is generally observed at moderate to high doses [25,29,31,32,33,34,35]. One study at 2 mg/L (20^6^ ng/L) found hypoactivity [12], but to our knowledge, this is the first study within a ng/L range, which may account for the diverging effect. Few studies have examined F1, and none at low doses. At moderate doses, Chen et al. observed the exact pattern that we observed of hyperactivity in the dark and hypoactivity in the light [33]; hyperactivity was also observed in other studies [36]. In contrast to PFOA-lineage F2, transgenerational PFOS effects present as totally different from the F0 pattern. The differences in the structure of sulfonic and carboxylic acids are known to exert different effects [31]; this phenomenon appears to continue into the F2 generation.

Mixture-exposed F0 larvae were hyperactive, F1 were hypoactive, and F2 possessed a variable response to dark and light stimuli. Though PFOS was present in a higher concentration than PFOA, PFOS did not appear to overpower PFOA’s presence or drive the mixture results as the mixture endpoints were quite different from the PFOS endpoints. Mixtures are generally understudied. Despite PFOS and PFOA being two of the most thoroughly investigated individual PFAS chemicals, their mixture at human levels has not been well-studied for locomotor behavior. However, a complex mixture including both chemicals induced hyperactivity at putative human serum levels [32], though the presence of other chemicals likely influenced the outcome. Very high exposure to a >1 g/L (10^9^ ng/L) mixture of nine PFASs in equal amounts was associated with hypoactivity [12]. However, this concentration may have caused lethargy-inducing toxicity as the LC50 of a 1:1 ratio of PFOA:PFOS has been demonstrated at ~37 mg/L (37^6^ ng/L) in zebrafish [11]. Given that we are never exposed to a single PFAS alone, and PFAS mixtures have been measured in amniotic fluid [37,38], the lack of knowledge on mixtures across generations necessitates more research, especially at environmentally relevant levels.

Gene expression dysregulation was the most sensitive and persistent endpoint observed across all chemicals and generations. While PFASs are not directly genotoxic, they are known to cause transcriptomic changes [25,29,39]. As exposure to low concentrations of PFASs is understudied, we chose to explore the full transcriptome using RNA-seq rather than targeted expression analysis. This study may provide genes of interest for future biomarkers of effect and for targeted analysis in low-exposure schemes. We report multi- and transgenerational effects in gene expression. In fact, for every chemical, more genes were differentially expressed as the generations progressed. The F1 generation showed 2–4× more DEGs than F0. The F2 generation showed 2–3× more DEGs than F1 (and 8–9× more than F0) even in the absence of exposure in the F1 and F2 generations, suggesting epigenetic regulation of the transcriptome. The affected pathways ranged from the immune system, xenobiotic metabolism, and steroid metabolism and synthesis (PFOA) to movement disorders and bone mineral density (PFOS). Surprisingly, the mixture caused relatively few DEGs compared to the single chemicals alone. In general, each chemical was associated with a unique set of DEGs in each generation. However, common to all chemicals in the F1 generation was the dysregulation of *si:dkey-14d8,* and to the F2 generation, *wbp2nl*. Little is known about the *Danio rerio* gene *si:dkey-14d8*; it is predicted to be involved in collagen fibril organization [40]. The F2 gene *wbp2nl* encodes a sperm protein that promotes oocyte fertilization [41]. This gene was upregulated by PFOS and mixture exposure (log2 fold change 1.20, 0.85, respectively) but downregulated by PFOA exposure (log2 fold change −1.23). In line with our null findings of changes in fecundity, *WBP2NL* expression was not associated with reproductive outcomes in a human study seeking prognostic fertilization factors [42]. *Wbp2nl* is silenced during early development [43]; thus, its activation in the F2 larvae by PFOS and the mixture may indicate aberrant epigenetic programming. While *wbp2nl* is mainly expressed by sperm, it is also found in the breast and kidney [44]—areas known to be PFAS targets [45,46]. It will be interesting for future studies to further phenotypically anchor the diverse transcriptomic pathways of each chemical and the mixture and establish biomarkers of effect for PFAS exposure.

In PFOA-exposed F0 larvae, *rpe65a* and *atp6v0e1* were downregulated. Downregulation of *rpe65a* is associated with retinal degeneration in zebrafish [47], and loss of RPE65 function leads to blindness in humans [48]. *Atp6v0e1* is involved in visual–motor behavior [49]. The downregulation of optical-related *rpe65a* and *atp6v0e1* may have contributed to the hypoactivity we observed in PFOA-exposed F0 larvae. In the F1 generation, xenobiotic pathways predominated, driven by the upregulation of *cyp3a7*. PFASs are known xenobiotic inducers of PXR and CAR pathways in humans and rodents; in zebrafish, these receptors have been shown to be unresponsive to PFOA [50]. However, as these data seek to inform human health, the change in *cyp3a7* expression implicating PXR and CAR activation is still meaningful. Additionally, *CYP3A7* in humans is enriched in fetal liver [51], underscoring the relevance of the embryonic zebrafish exposure model to human developmental health. Another upregulated molecule in xenobiotic pathways was *dusp16*, which has a role in immune function [52]. Though immune dysregulation does not feature prominently in the PFOA F1 pathway profile, PFASs are a demonstrated immunotoxicant [53], and the *dusp* genes appeared in DEGs of the F0 generation and additionally in the pathway analysis of the F2 generation. In addition to immune system pathway disruption in the F2 generation, steroid synthesis was affected, and the glucose-homeostasis-related pancreatic gene *amy2al2* was the most upregulated DEG at every concentration. PFOA has been shown to increase steroid hormone levels in zebrafish larvae [54], and links between PFOA serum levels and diabetes risk have been established in humans [55,56,57]. Immune dysfunction appears to be a significant outcome of low-level PFOA exposure, though effects may not be seen until later generations.

In PFOS-exposed F0 larvae, *irs2a* and inflammatory response gene *irg1l* were upregulated, and the kinesin gene *kif3c* was downregulated. Besides its known glucose metabolism function [56,57,58], *irs2a* has an emerging function in hypoxia protection [59,60]. Hypoxia and inflammation, in combination with decreased expression of the photoreceptor *kif3c* gene [61], may have contributed to the hypoactivity we observed in PFOS-exposed larvae. Others have found downregulation of the histamine H1 receptor [32] and steroidogenic enzymes [29,62] at moderate to high exposure. Relatively few genes were differentially expressed in the F0 generation as compared to the F1 and F2 generations. In the F1 generation, DNA-binding genes *satb1a* and *npas4a* were two of the most upregulated genes. *Satb1a* and *npsa4a* expression is localized to the CNS in larvae [63,64]. In line with the present behavioral results of hyperactivity from very low exposure, *npas4* expression is increased in response to neuronal activity [64]. Less is known about *satb1a* in zebrafish. In humans, *SATB1* remodels chromatin in thymocyte differentiation into T-cells [65,66]. Pathway analysis results included lowered chemotaxis of immune cells, increased steroid synthesis, survival of neuronal cell types, and movement disorders. F1 larvae were the only group across all chemicals and generations where the direction of behavior (hyper- or hypoactivity) had no agreement between light and dark conditions. The upregulation of neuronal activity gene *npas4* may have contributed to hyperactivity in the dark; the movement disorder pathway is one molecular indication of the contrasting responses to light and dark. Other studies in zebrafish have not examined and compared for gene expression with F1-lineage behavior. As in F1, F2 larvae showed increased pathways involving steroids and, additionally, other lipids. The pancreatic gene *cela1.5* was the most upregulated DEG at ultra-low and very low exposures and was moderately upregulated at low exposure. Lipid metabolism disruption has been previously linked to PFOS exposure at moderate levels in F0 fish [67], but we did not observe this effect until the F2 generation. Bone mineral density and other connective tissue pathway disruptions were also a PFOS F2-specific occurrence. Increased lipid pathways and decreased connective tissue pathways do not seem to explain the observed hyperactivity in the F2 generation; however, a non-specific movement disorder pathway was also increased. More research is required in F2 larvae to fully understand the scope of the ancestral effects of PFOS exposure. In most generations, PFOS exposure was associated with pathways of increased lipid synthesis, which complements the thoroughly-studied PFOS-associated high cholesterol in humans [68,69,70].

Mixtures are a rapidly expanding field of research, and low levels are highly relevant to human health. In mixture-exposed larvae, few DEGs were expressed in all generations compared to individual PFASs (Figure 2). The F0 generation exhibited dysregulation of genes involved in basic cellular processes, with no obvious influence on the observed hyperactivity. Similarly, in their assessment of behavior and gene expression in a complex mixture including PFOS and PFOA, Khezri et al. [30] could not rationalize a clear link between exposure-associated hyperactivity and DEGs. More research is certainly needed to elucidate the complex transcriptomic dynamics underpinning behavioral outcomes in mixtures. The F1 generation showed dysfunction in pancreatic genes *zgc:92590*, *cela1.5,* and *cpa4*. F1 downregulation in optic-related gene *rlbp1b* [71] could have contributed to the observed hypoactivity of larvae in light and dark. F2 larvae downregulated the muscular gene *tcap*, yet upregulated growth genes *fzd6* and *fgfbp2*. When significant DEGs from all generations were collated, pathway analysis revealed immune dysfunction and developmental deficits predicting organismal and cell death; however, mortality was unaffected in any generation. The present results suggest mixture exposure does not cause overt harm in any generation; however, the transcriptome of developmentally exposed fish may be an early indicator of latent embodied effects. Perhaps a longer experiment with aged fish would reveal mixture-associated latent mortality.

Egg production in females was measured to estimate fecundity in the F0 and F1 generations. Changes in fecundity may have implications for reproductive health, the offspring, as well as the ecosystem. In humans, there is no consensus on fecundity and PFAS exposure, possibly owing to the multiple ways of defining fecundity in humans. Multiple epidemiological studies have found a decrease in fecundity with PFOA or PFOS [72,73,74,75], while some have found no effect in either [76,77]. There was no effect on the fecundity of F0 exposure to PFAS. Of note, controls in the PFOA F0 group produced significantly fewer eggs than controls in the PFOS (*p =* 4 × 10^−3^) but not mixture (*p =* 0.15) groups (one-tailed *t*-test) (PFOS and mixture controls were not significantly different (*p =* 0.22, two-tailed *t*-test)). When egg production in PFOA-exposed larvae was compared to control data from the PFOS and mixture larvae, there was actually a significant decrease in egg production at low exposure (*p =* 2.1 × 10^−2^) (data not shown). Decreased zebrafish egg production was observed in another study on low-level exposure to PFOA [37] and at moderate exposure in the crustacean *Daphnia magna* [78]; however, in wild-caught fish, hepatic levels of PFOA had no association with fecundity [79]. In the F1 generation, very low exposure lineage fish produced significantly fewer eggs. In the only other transgenerational study of fecundity, Marziali et al. [80] found no effect in F0–F2 in harlequin flies. In PFOS studies, fecundity is found to be either decreased at moderate doses [78,81] or to have no effect [34,77], including no effect in F1 and/or F2 [34,78]. In an F0–F2 study of a moderate dose PFAS mixture containing low doses of PFOA and PFOS on Japanese medaka, Lee et al. [82] reported no significant effect. Overall, the present results suggest little to no effect on fecundity in F0–F1 zebrafish exposed to low levels of PFAS.

Sex determination in laboratory zebrafish is polygenic and is thought to be influenced by their environment, which can include exposure to contaminants [83]. Alterations in the male:female ratio are thus a common endpoint in endocrine disruption studies, with a shift in either direction indicating disruption. In the F0 generation, there was no significant change in sex ratio following exposure. In the F1 generation, the very low level of the mixture and every concentration of PFOA caused an increase in the ratio (significantly more males). The authors note the abnormal lack of males in the PFOA control group, which may have led to a false-positive result. Additionally, due to a lack of access to research animals during the SARS-CoV-2-related institutional shutdown, only one cohort of PFOA F1 and two cohorts of mixture F1 fish could be observed. No changes were observed in the PFOS-exposed sex ratio in either generation. Other studies have observed a decreased ratio following F0 PFOS exposure [34]. Exposing the F0 and F1 generations to a mixture of four PFAS, including PFOA and PFOS, did not result in any shift [82]. The scarcity of replicates for all groups and abnormal PFOA control fish do not allow meaningful conclusions to be drawn from this endpoint in the present study.

As each generation was differentially affected by each chemical, it is pertinent to summarize the similarities and differences of each chemical’s discussed effects in the F0, F1, and F2 generations separately. Additionally, human health research and policies are mainly concerned with the directly exposed subject. In the F0 generation, PFOA exposure was associated with hypoactivity, with a potential visual–motor impact occurring via the downregulation of vision-related genes *rpe65a* and *atp6v0e1* [47,49]. Similarly, PFOS-exposed larvae were also hypoactive in the light and had a downregulated photoreceptor gene (*kif3c*) [61]. Ophthalmic health should be observed more closely in future studies examining behavior response to visual cues, especially as zebrafish eyes are in constant contact with the exposure solution. The mixture-exposed larvae were, in contrast, hyperactive and showed no clear disruption of a particular pathway, dysregulating instead the genes involved in basic cellular processes. No chemical was associated with a significant change in adult fecundity, body weight, length, or sex ratio. In the F1 generation, PFOA exposure was associated with hyperactivity and xenobiotic response. PFOS-related behavior varied by light or dark status in the only locomotor disagreement in the study; upregulated CNS-related genes could account for the hyperactivity in the dark. The mixture larvae displayed hypoactivity and dysfunctional pancreatic genes. Additionally, the downregulation of optic-related gene *rlbp1b* [71] in the mixture larvae could complicate behavior results, as in the F0 generation. Overall, each chemical was associated with disparate pathways in the F1 generation, in line with different behavioral patterns across the chemicals. No chemical was associated with a reliable change in adult fecundity or sex ratio. In the F2 generation of PFOA- and PFOS-exposed larvae, pancreatic genes were most affected, likely leading to the observed alterations in hormone-related pathways. Additionally, immune pathways were affected in the PFOA and mixture groups. Each chemical in the F2 generation was associated with a different behavioral pattern (PFOA: hypoactivity; PFOS: hyperactivity), with the mixture showing both hyper- and hypoactivity. In sum, the evidence points to varying effects of PFASs depending on both the specific chemical and degree of exposure.

This study provides the first report on the multigenerational effects of environmental-level PFOA exposure on zebrafish behavior and of a mixture of the two chemicals on behavior and gene expression. Further, it is the first report of the transgenerational effects of PFOA, PFOS, and a 1:1 mixture in terms of behavior and transcriptomics. The next steps in this line of research will be to examine the epigenetic influences set in motion by these PFASs. Effects onto the F2 generation have been reported in PFBS [84] and PFOS-alternative F-53B exposure [85] at moderate levels. Intriguingly, low-level PFAS exposure in the present study continued to exert effects generations after exposure cessation. Gene expression dysregulation increased as the generations progressed, with F2 exhibiting far more DEGs than F0, suggesting epigenetic regulation of expression in the absence of a chemical stressor. In general, the DEGs in each generation and in each chemical had little overlap. Interestingly, the mixture had a relatively small influence on the number of DEGs compared to the individual PFASs. The unique suites of DEGs underscore the differential effects of different functional groups of PFASs and individual PFASs versus a mixture and suggest different mechanisms of action in the production of the observed transcriptomic signatures and behavioral phenotypes.

It is the authors’ aim that these results inform decision-making regarding safe contaminant limits in drinking water, food sources, and aquatic habitats. Future studies into the mechanisms of epigenetic dysregulation under exposure will be of great interest. PFAS replacements, including “short-chain” alternatives to PFOA and PFOS, will be critical to study as well, both individually and in environmentally relevant mixtures.

## Figures and Tables

**Table 1:** Endpoints of PFAS exposure in zebrafish (*Danio rerio*) across all chemicals, concentrations, and generations. Survival, morphological abnormalities, swim distance, fecundity, sex ratio: percent change. DEGs: number. Blue: decreased endpoint. Orange: increased endpoint. Grey: both increased and decreased endpoints.

**F0 Generation Endpoint**	**Concentration**	**PFOA**	**PFOS**	**Mixture**
Survival	Ultra-low			
	Very low			
	Low			
Morphological abnormalities	Ultra-low			
	Very low			
	Low			
Swim distance (dark)	Ultra-low	−10.5%		+3.7%
	Very low	−10.2%		+3.6%
	Low	−4.2%		+12.1%
Swim distance (light)	Ultra-low	−11.6%		+9%
	Very low	−18.8%	−8.16%	+9.7%
	Low		−5.4%	+16%
Differentially-expressed genes	Ultra-low		1	6
	Very low	1	54	
	Low	14		2
Fecundity	Ultra-low	+85%		
	Very low	+42.7%		
	Low			
Sex ratio (% males)	Ultra-low			
	Very low			
	Low			
**F1 Generation Endpoint**	**Concentration**	**PFOA**	**PFOS**	**Mixture**
Survival	Ultra-low			
	Very low			
	Low	+26.7		
Morphological abnormalities	Ultra-low			
	Very low			
	Low	−7.4%		
Swim distance (dark)	Ultra-low		+15.4%	−12.2%
	Very low	+4.6%	+10.2%	−9.9%
	Low	+9%		−12%
Swim distance (light)	Ultra-low			−15.5%
	Very low	+9.6%		
	Low	+10.6%	−10.6%	
Differentially-expressed genes	Ultra-low	17	5	35
	Very low	106	2	12
	Low	49	149	7
Fecundity	Ultra-low			
	Very low			
	Low		−28.2%	
Sex ratio (% males)	Ultra-low	+30.2%		
	Very low	+55.4%		+28.9%
	Low	+57.1%		
**F2 Generation Endpoint**	**Concentration**	**PFOA**	**PFOS**	**Mixture**
Survival	Ultra-low			
	Very low			
	Low			
Morphological abnormalities	Ultra-low			
	Very low			
	Low			
Swim distance (dark)	Ultra-low	−8.8%		−3.8%
	Very low		+9.4%	+11.2%
	Low	−7.3%		
Swim distance (light)	Ultra-low			−14.5%
	Very low	−4.7%	+8.8%	
	Low	−14.8%		
Differentially-expressed genes	Ultra-low	112	484	69
	Very low	106	23	1
	Low	302	7	9

**Table 2:** Top 5 up- and downregulated DEGs in each chemical, concentration, and generation of zebrafish (*Danio rerio*) and the pathways affected.

Gen.	Chemical	Conc.	Upregulated	Downregulated	Pathways
F0	PFOA	Ultra-low	NA	NA	
		Very low	NA	*rpe65a*	
		Low	*ENSDARG00000075180*, *gabarapl2*, *capzb*, *npc2.1*, *dusp1*	*atp6v0e1*, *trim36*, *phtf2*,	
	PFOS	Ultra-low	*irs2a*	NA	
		Very low	*irg1l*, *si:ch211-153b23.4*, *psma5*, *si:dkeyp-1h4.8*, *si:ch211-153b23.5*	*kif3c*, *ENSDARG00000087345*, *map4k3b*, *prodha*, *ca4a*	
		Low	NA	NA	NA
	Mixture	Ultra-low	NA	*fkbp9*, *si:ch211-251f6.6*, *hbbe1.3*, *ENSDARG00000092364*, *ENSDARG00000088687*	
		Very low	NA	NA	
		Low	*slc6a19a.1*, *entpd8*	NA	
F1	PFOA	Ultra-low	*dusp16*, *pycr1b*, *tmigd1*, *wbp2nl*, *serpina7*	*zgc:136410*, *lgals1l1*, *pkhd1l1.2*, *pcnx3*, *si:ch211-125e6.5*	NA
		Very low	*dusp27*, *gadd45ba*, *lims2*, *asb2b*, *cuzd1.2*	*c3a.2*, *c4b*, *mthfd1l*, *lgals1l1*, *si:ch211-125e6.5*,	Xenobiotic metabolism, estrogen receptor signaling
		Low	*tmigd1*, *npas4a*, *dusp16*, *gadd45ba*, *dusp27*	*trmt1*, *pitrm1*, *ercc6l*, *ifi44d*, *cdk16*	NA
	PFOS	Ultra-low	*satb1a*, *si:ch211-103n10.5*, *zgc:172051*, *spint1b*, *akap17a*	NA	NA
		Very low	*npas4a*	*slc43a2a*	NA
		Low	*zmat5*, *ENSDARG00000082716*, *slc9a2*, *dusp19b*, *gadd45bb*	*mfsd14ba*, *ggt5b*, *ppp6r2b*, *dennd5a*, *nkx3.3*	Lipid metabolism, cell death
	Mixture	Ultra-low	*zgc:92590*, *smyhc2*, *amy2a*, *calcoco1b*, *si:dkey-14d8.7*	*smtnl*, *fh*, *panx1a*, *trak2*, *g6pc1a.1*	NA
		Very low	*cela1.3*, *si:dkey-14d8.7*, *amy2a*, *si:ch211-240l19.8*, *pla2g1b*	*panx1a*	NA
		Low	*cpa4*, *zgc:92590*, *hsd11b2*, *pla2g1b*, *si:dkey-14d8.7*	*ms4a17a.8*, *rlbp1b*	NA
F2	PFOA	Ultra-low	*amy2al2*, *glg1a*, *slc17a6a*, *actl6a*, *ENSDARG00000096135*	*ms4a17a.8*, *tfdp2*, *prss59.2*, *srsf5b*, *LOC100538179*	Mitochondrial membrane potential, organismal injury
		Very low	*amy2al2*, *crp2*, *LOC103910030*, *eef2k*, *pcnp*	*scn2b*, *cela1.3*, *cela1.5*, *tmem97*, *LOC101882496*	Cholesterol and other sterol synthesis
		Low	*amy2al2*, *LOC103910030*, *irg1l*, *eef2k*, *si:ch211-260e23.9*	*lhx2b*, *smc1a*, *LOC110439320*, *rlbp1b*, *ms4a17a.8*	Immune cell function and trafficking, cell death, glucose homeostasis
	PFOS	Ultra-low	*cela1.5*, *haao*, *ENSDARG00000115830*, *atp9b*, *ENSDARG00000097916*	*pgk1*, *si:ch211-260e23.9*, *crtac1a*, *cyp8b1*, *rrm2*	Steroid synthesis, bone mineral density, connective tissue
		Very low	*cela1.5*, *lhx2b*, *cela1.3*, *mafb*, *zmp:0000001048*	*b3gntl1*, *si:ch211-196h16.5*, *arpc5a*, *rbm4.1*, *bnip4*	NA
		Low	*ENSDARG00000115830*, *LOC100536187*, *pcdh1b*, *smdt1a*	*cfp*, *ddx47*, *LOC108179091*	NA
	Mixture	Ultra-low	*fzd6*, *gatm*, *bub3*, *fgfbp2b*, *il20ra*	*tcap*, *mmp9*, *bnip4*, *pfkfb3*, *si:dkey-85k7.7*	NA
		Very low	*purab*	*hbae5*, *c4b*, *hbae1.3*, *cebpa*	NA
		Low	*fgfbp2b*, *si:dkey-102c8.3*	*si:ch211-281l24.3*, *anxa1c*, *si:ch211-240l19.8*, *calcoco1b*, *c4b*	NA

**Table 3:** Significant DEGs involved in epigenetic processes in the F2 generation of zebrafish (*Danio rerio*).

Chemical	Gene Symbol	log2FC	*p*-Value	Function
PFOA	*actl6a*	1.7	0.0018	Chromatin modifying
	*foxa3*	1.15	0.0067	HAT recruitment
	*glyr1*	0.94	0.0085	Nucleosome activity
	*kdm3b*	0.99	0.0080	Histone lysine demethylase
	*mat1a*	−1.6356	0.0035	Methionine adenosyltransferase
	*max*	−1.24	0.0043	HMT interaction
	*sap30l*	−1.18	0.0001	HDAC subunit
	*smc1a*	−1.93	0.0000	Chromatid tethering
	*ybx1*	−1.33 (ultra-low); 1.06 (low)	<0.004	DNA binding
PFOS	*chmp2a*	−0.91	0.0079	Chromatin modifying
	*h1-0*	0.91	0.0028	H1.0 linker histone
	*hbp1*	0.97	0.0017	DNMT1 repressor
	*hmg20a*	0.89	0.0085	HMT recruitment
	*hmgn2*	−1.06	0.0046	Chromatin modifying
	*hnrnpk*	−1.19	0.0019	ssDNA binding
	*kdm1a*	−0.87	0.0071	Lysine demethylase 1A
	*meaf6*	−1.4	0.0013	HAT interactor
	*prdm9*	−1.41	0.0053	HMT recrutiment
	*riox2*	1.48	0.0004	HDMT
	*setd5*	0.96	0.0020	KMT2E paralog
	*tox2*	1.26	0.0001	Chromatin modifying
	*usf1*	−1.01	0.0071	Chromatin modifying
Mixture	*h2bc1*	−0.95	0.0006	H2B clustered histone 1

**Table 4:** Pathway analysis (IPA) of all DEGs from each concentration and generation of mixture-exposed zebrafish (*Danio rerio*) larvae combined.

Rank	Diseases or Functions Annotation	*p*-Value	Bias-Corrected z-Score	# Molecules
1	Organismal death	3.59 × 10^−3^	1.714	22
3	Morbidity or mortality	1.81 × 10^−3^	1.429	23
10	Quantity of cytokine	3.53 × 10^−3^	0.834	5
11	Infiltration by neutrophils	1.30 × 10^−3^	0.793	5
12	Cell movement of neutrophils	3.56 × 10^−3^	0.751	6
18	Necrosis	6.16 × 10^−3^	0.603	23
24	Chemotaxis of leukocytes	9.40 × 10^−4^	0.307	7
27	Quantity of myeloid cells	1.41 × 10^−3^	0.301	9
41	Cellular infiltration by phagocytes	2.65 × 10^−3^	−0.026	6
42	Cellular infiltration by myeloid cells	4.04 × 10^−3^	−0.028	6
46	Cellular infiltration by leukocytes	1.09 × 10^−3^	−0.144	8
57	Accumulation of leukocytes	5.69 × 10^−3^	−0.402	5
87	Inflammatory response	5.20 × 10^−3^	−1.872	10

## Supplementary Materials

The following supporting information can be downloaded at: https://www.mdpi.com/article/10.3390/toxics10060334/s1. Figure S1: Schematic of experimental design for obtaining embryos and for fecundity assay. Figure S2: Box plots of F0 and F1 fecundity data. Table S1: Replicates and *n*-values for all experiments. Table S2: Endpoint data and significance reporting for survival, abnormalities, behavior, fecundity, and sex ratio. Table S3: F0 DEGs. Table S4: F1 DEGs. Table S5: F2 DEGs. Table S6: IPA summaries. File S1: Custom R code for analysis of behavioral data.
