# Supplementary material for: Multi- and Transgenerational Effects of Developmental Exposure to Environmental Levels of PFAS and PFAS Mixture in Zebrafish (Danio rerio)
Source: Toxics. 2022 Jun 18;10(6):334. doi: 10.3390/toxics10060334 (PMC9228135; doi:10.3390/toxics10060334)
Supplement: Supplementary file 1 [file toxics-10-00334-s001.zip › File S1_Custom R code_behavioral analysis.pdf]

```
# Clear working space
rm(list = ls())

# Install packages for the people use the packages first time
# Load packages
library(tidyverse)
library(readxl)
library(lubridate)
library(plotly)
library(ggpubr)

#1. Load the excel files and name the column head
#1.1 Make a function to read all Excel spreadsheets
LA_read <- function(x){
  dat <- read_excel(x, col_names = FALSE, skip = 4)
  names(dat) <- c("Treatment", "Plate", "Location", "Time",
                "err%","Distance", "Velocity", "Turn_angle",
                "Angular_Velocity")
  return(dat)
}

#1.2 List all file names with .xlsx in the folder
file_vec <- list.files(pattern = "xlsx$")
file_vec

#1.3 Use the LA_read function to load the PFOA excel
#### spreadsheets for analysis and use map_dfr to combine
#### all dataframes
dat_all <- map_dfr(file_vec,
                 LA_read, .id = "Parent")

# Check treatment names
unique(dat_all$Treatment)

#2. Clean up data
##2-1. Define light and dark cycles
dat_all2 <- dat_all %>%
  # Manipulate the time index
  separate(Time, into = c("Start", "End"), sep = "-") %>%
  mutate(End = hms(End)) %>%
  mutate(Sec = as.numeric(End - hms("00:00:00"))) %>%
  select(-End) %>%
  group_by(Parent, Location, Treatment) %>%
  mutate(CycleN = (Sec %/% 30 - 1) %/% 6) %>%
```

```
  mutate(Cycle = ifelse(CycleN %% 2 == 0, "Light", "Dark"))

# Replace "Start" to "0:00:00"
dat_all3 <-dat_all2 %>%
  ungroup() %>%
  mutate(Start = recode(Start, "Start" = "0:00:00"))


#3. Assign sample ID
dat_all4 <- dat_all3 %>%
  mutate(Sample_ID =
         group_indices(., Parent, Treatment,
                Plate, Location))


#4. Separate dark and light data
# Filter light data
dat_light <- dat_all4 %>%
  filter(Cycle == "Light")

# Filter dark data
dat_Dark <- dat_all4 %>%
  filter(Cycle == "Dark")

#5. Quality control: Use outlier methods to remove the abnormal data
# 5-1. Remove the outliers from light cycle
# Filter control light data
dat_light_control <- dat_light %>%
  filter(Treatment == "control")

# Calculate the IQR of light cycles in control data
light_q25 <- quantile(dat_light_control$Distance, 0.25)
light_q75 <- quantile(dat_light_control$Distance, 0.75)
IQR <- light_q75 - light_q25
upper_L <- light_q75 + IQR
upper_L_C <- light_q75 + 1.5*IQR


# Starting from the third data points, remove the series that have
# two serial data points larger than upper bound in the light cycles
dat_C_light <- dat_light %>%
  group_by(Sample_ID) %>%
  filter(!any((Distance[3:24] > upper_L_C) &
         (lead(Distance[3:24]) > upper_L_C))) %>%
```

```
  ungroup()

# Check how many data series are removed
n_distinct(dat_light$Sample_ID)-n_distinct(dat_C_light$Sample_ID)

# After removing the light data series, remove the associated dark series
dat_C_Dark <- dat_Dark %>%
  semi_join(dat_C_light, by = c("Sample_ID"))

# Make a new data from the list that has completed the first QC step
dat_C <- bind_rows(dat_C_light, dat_C_Dark)

# 5-2. Remove the abnormal data from dark cycle:
# Calculate the median distance of light cycles
Dark_upper <- median(dat_C_light$Distance)

# Remove dark cycle data series if two serial data points are lower
# than the median of light cycles
dat_C2_Dark <- dat_C_Dark %>%
  group_by(Sample_ID) %>%
  filter(!any((Distance < Dark_upper) &
              (lead(Distance) < Dark_upper))) %>%
  ungroup()

# After removing the dark data series, remove the associated light series
dat_C2_light <- dat_C_light %>%
  semi_join(dat_C2_Dark, by = c("Sample_ID"))

# Make a new datafrom that have completed the 2nd QC step
dat_C2 <- bind_rows(dat_C2_light, dat_C2_Dark)

# Check how many data series are removed at the 2nd QC step
n_distinct(dat_C_light$Sample_ID)-n_distinct(dat_C2$Sample_ID)

# 5-3. Figure which data series have similar light and dark mean distance
dat_filter <- dat_C2 %>%
  group_by(Sample_ID, Cycle) %>%
  summarize(mean_dis = mean(Distance)) %>%
  spread(Cycle, mean_dis) %>%
  mutate(Ratio = Light/Dark) %>%
  filter(Ratio > 0.9)

# Remove the data series that have similar light and dark mean distance
dat_C3 <- dat_C2 %>%
```

```
  anti_join(dat_filter, by = "Sample_ID")

# Check how many data series are removed at the 3rd QC step
n_distinct(dat_C2$Sample_ID)-n_distinct(dat_C3$Sample_ID)

# Check how many data series are removed from all QC step
n_distinct(dat_light$Sample_ID)-n_distinct(dat_C3$Sample_ID)

# 5-4. Record which samples were removed by comparing the new and
###### old data sheets
remove_list <- dat_all4 %>%
  anti_join(dat_C3, by = c("Sample_ID"))

# Save the remove list into a data frame
remove_list_save <- remove_list %>%
  distinct(Parent, Treatment, Location, Plate, Sample_ID)

# Save the datasheet into a csv file
write.csv(remove_list, "Initial-remove_list.csv", row.names = FALSE)

## 6. Plot the behavior plots after QC
# Calculate the statistics of each treatment
dat_C3_Combine <- dat_C3 %>%
  group_by(Start, Treatment) %>%
  summarise(N = n(),
        Mean_Distance = mean(Distance),
        SD_Distance = sd(Distance),
        SE = SD_Distance/N) %>%
  ungroup() %>%
  mutate(Start = as.numeric(hms(Start))/60)

# Define the shade region in a different dataframe
rect_C3 <- data.frame(
  xstart = c(3, 9, 15, 21),
  xend = c(6, 12, 18, 24)
)

# Plot
Combine <- ggplot() +
  geom_rect(data = rect_C3, aes(xmin = xstart, xmax = xend,
                      ymin = -Inf, ymax = Inf),
        alpha = 0.2) +
  geom_line(data = dat_C3_Combine,
        aes(x = Start, y = Mean_Distance,
```

```
            group = Treatment), size = 0.8) +
  geom_point(data = dat_C3_Combine,
          aes(x = Start, y = Mean_Distance,
             fill = Treatment), shape = 21, color = "black",
          size = 5) +
  scale_fill_brewer(type = "seq", palette = "Oranges", direction = 1) +
  scale_x_continuous(breaks = seq(0, 24, by = 3)) +
  theme_classic() +
  theme(legend.position = "none",
      axis.title = element_text(size = 16),
      axis.text = element_text(size = 16)) +
  xlab("Time (min)") +
  ylab("Distance moved (cm)")


#7. Summarize the distance data after and before QC:
# After QC
Summary_Distance <- dat_C3 %>%
  group_by(Treatment, Cycle) %>%
  summarise(
    N = n()/24,
    mean = mean(Distance, na.rm = TRUE),
    sd = sd(Distance, na.rm = TRUE),
    CV = sd/mean,
    se = sd/(N^0.5)
  )


#8. Run ANOVA and Tukey pairwise to compare the distance
#8-1. After QC- dark data
dat_C3_Dark <- dat_C3 %>%
  filter(Cycle == "Dark")

# Run ANOVA
Dark.aov <- aov(Distance ~ Treatment, data = dat_C3_Dark)

# Check ANOVA results
summary(Dark.aov)

# Check Tukey pairwise results
TukeyHSD(Dark.aov)

#8-2. After QC- light data
dat_C3_Light <- dat_C3 %>%
```

```
  filter(Cycle == "Light")

# Run ANOVA
Light.aov <- aov(Distance ~ Treatment, data = dat_C3_Light)

# Check ANOVA results
summary(Light.aov)

# Check Tukey pairwise results
TukeyHSD(Light.aov)


#9. Plot bar plot
#9-1 (1) Define the significant treatment in a dataframe: ex. 70ng/L and 7ng/L
#### were significant different from control;
#### (2) Recode the treatment label
Summary_Distance_text <- Summary_Distance %>%
  mutate(Sig = ifelse(Treatment %in% c("70ng/L", "7ng/L"),
                 "***", NA)) %>%
  ungroup() %>%
  mutate(Treatment = recode(Treatment,
                       "control" = 0,
                       "7ng/L" = 7,
                       "70ng/L" = 70,
                       "700ng/L" = 700)) %>%
  mutate(Treatment = factor(Treatment))

#9-2. Make the bar plot
barplot <-
ggplot(Summary_Distance_text, aes(x = Cycle,
                        y = mean,
                        fill = Treatment)) +
  geom_col(position = "dodge", colour = "black") +
  annotate(xmin = 0.5, xmax = 1.5, ymin = -Inf, ymax = Inf,
         alpha = 0.2, geom = 'rect') +
  geom_errorbar(aes(ymax = mean + se,
              ymin = mean - se),
             position = position_dodge(.9),
             width = 0.2) +
  geom_text(aes(y = mean + se + 0.3, label = Sig),
         position = position_dodge(.9),
         size = 6) +
  scale_fill_brewer(name = "PFOA \nconcentration (ng/L)",
              type = "seq",
```

```
                  palette = "Oranges", direction = 1) +
  xlab("") +
  ylab("Distance moved (cm)") +
  theme_classic() +
  theme(panel.grid = element_blank(),
        axis.title = element_text(size = 16),
        axis.text = element_text(size = 16))


#10. Combine the two figures with one row (nrow = 1)
figure <- ggarrange(Combine, barplot, nrow = 1)

#11. Save the figure.
###  Check the png file, modify the height and width if needed
ggsave("example.png", figure, height = 6, width = 16)
```
