# Supplementary figures and images for: Multi- and Transgenerational Effects of Developmental Exposure to Environmental Levels of PFAS and PFAS Mixture in Zebrafish (Danio rerio)
Source: Toxics. 2022 Jun 18;10(6):334. doi: 10.3390/toxics10060334 (PMC9228135; doi:10.3390/toxics10060334)

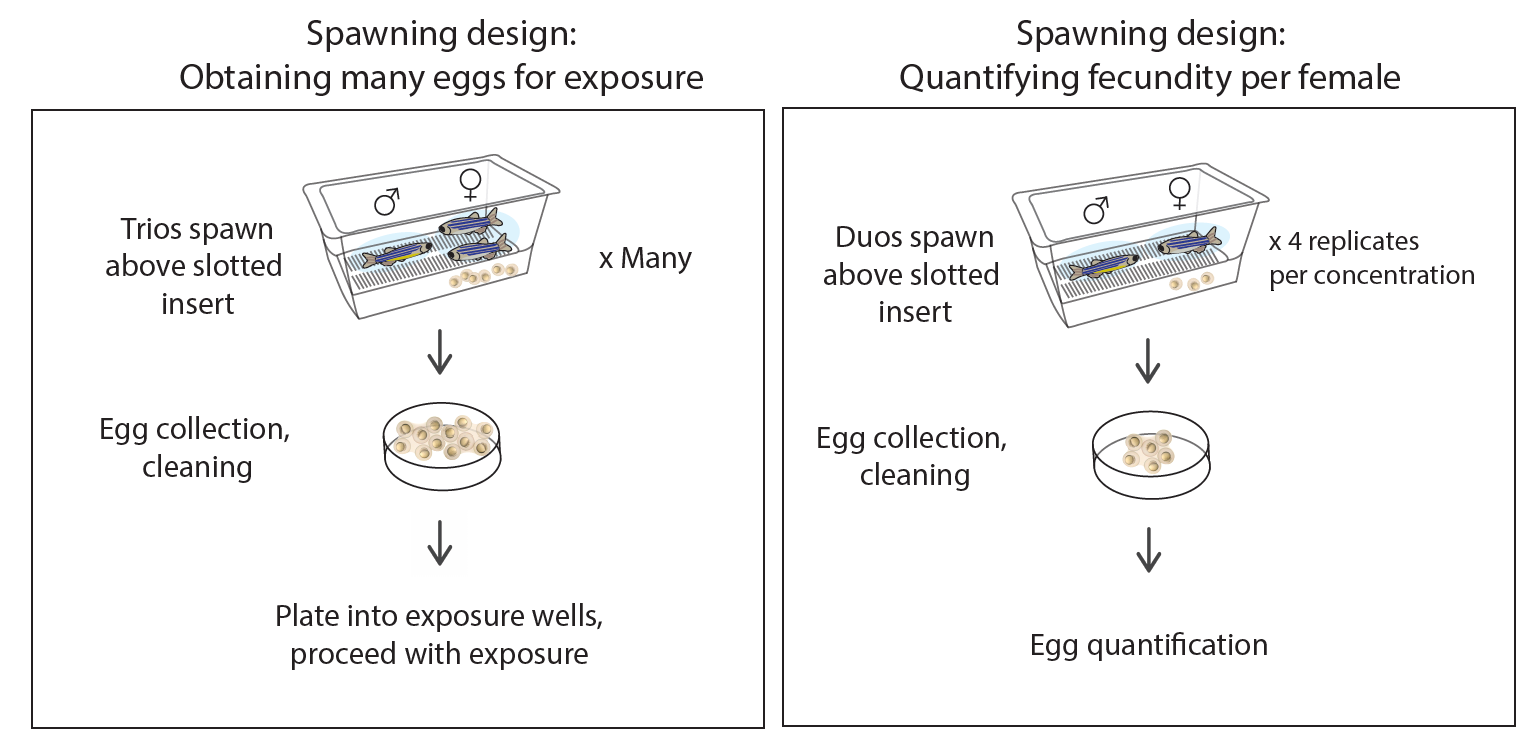

Supplement: Supplementary file 1 [file toxics-10-00334-s001.zip › Figure S1_breeding and fecundity comparison experimental design.PNG]

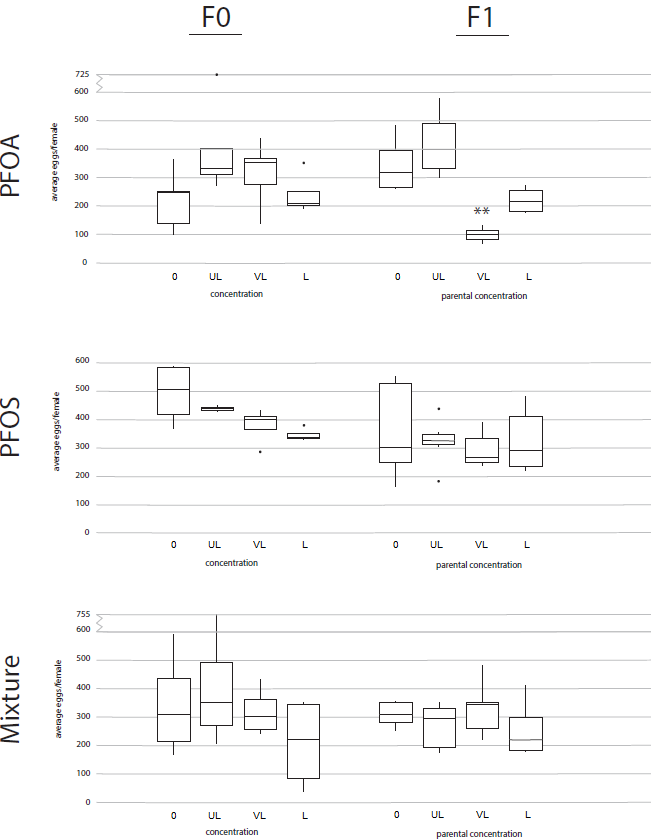

Supplement: Supplementary file 1 [file toxics-10-00334-s001.zip › Figure S2_fecundity graphs.png]
